# Nonlinear association between gamma-glutamyl transferase to high-density lipoprotein cholesterol ratio and risk of progression from normoglycemia to prediabetes: a 5-year cohort study

**DOI:** 10.3389/fendo.2025.1552044

**Published:** 2025-07-07

**Authors:** Chuang Gao, Cailing Yu, Peijie Shi, Dehong Liu, Qiming Li, Yong Han

**Affiliations:** ^1^ Department of Emergency, Shenzhen Dapeng New District Kuichong People’s Hospital, Shenzhen, China; ^2^ Department of Emergency, Shenzhen Second People’s Hospital, Shenzhen, Guangdong, China

**Keywords:** prediabetes, γ-glutamyl transferase, γ-glutamyl transferase to high-density lipoprotein ratio, nonlinearity, competing risk model

## Abstract

**Objective:**

Current research on the association between the Gamma-glutamyl transferase to high-density lipoprotein ratio (GHR) and the risk of prediabetes (pre-DM) remains scarce. This study aims to explore the potential link between GHR and the risk of progression from normoglycemia to pre-DM.

**Methods:**

This retrospective cohort study included 8,168 individuals who voluntarily underwent health examinations at Shenzhen Dapeng New District Kuichong People’s Hospital between January 2018 and December 2023. To assess the association between GHR and the risk of developing pre-DM, Cox proportional hazards regression models were employed. Cox proportional hazards regression model with cubic spline function was further utilized to investigate potential nonlinear association. Moreover, a competing risk Cox proportional hazards model was applied to account for the progression from normoglycemia to diabetes (DM) as a competing event in the progression from normoglycemia to pre-DM. Subgroup analyses and multiple sensitivity analyses were also performed to ensure the robustness of the findings.

**Results:**

Following multivariate adjustment, elevated GHR demonstrated a significant correlation with increased risk of progression from normoglycemia to pre-DM, showing a hazard ratio(HR) of 1.061 (95% CI: 1.028-1.095) for each 5-unit increment. A nonlinear relationship between them was identified, with an inflection point at a GHR value of 24.37. On the left side of the inflection point, the HR for the association between GHR (per 5-unit increase) and pre-DM risk was 1.394 (95% CI: 1.197, 1.623). Furthermore, the competing risk model revealed an HR of 1.05 (95% CI: 1.02, 1.09) for the association between GHR (per 5-unit increase) and pre-DM risk. Multiple sensitivity analyses confirmed the stability and reliability of these results.

**Conclusion:**

This study demonstrates that elevated GHR exhibits both a positive and nonlinear relationship with the risk of progression from normoglycemia to pre-DM among Chinese adults. Maintaining GHR values below the threshold of 24.37, coupled with further reduction efforts, may serve as an effective strategy to minimize pre-DM risk.

## Introduction

Diabetes Mellitus (DM), a complex metabolic condition arising from the interplay between hereditary predisposition and environmental influences, manifests through compromised insulin action, inadequate hormone production, and disrupted glucose regulation (1). The escalating prevalence and substantial burden of DM-related morbidity and mortality have established it as a critical challenge to global health systems (2–5). The intermediate metabolic state known as prediabetes (pre-DM) represents a crucial phase where glycemic parameters exceed normal limits but fall short of DM diagnostic thresholds (6). This condition not only heightens DM risk but also independently contributes to various complications, including ocular damage, renal dysfunction, and adverse cardiovascular outcomes (7–10). Recent data from the International Diabetes Federation (IDF) revealed that 374 million adults globally exhibited pre-DM in 2017, constituting 7.7% of the population. Projections indicate this figure will surge to 548 million by 2045, reaching 8.4% worldwide (4). Within China specifically, epidemiological investigations utilizing American Diabetes Association (ADA) criteria have identified a remarkably high pre-DM prevalence of 35.7%, surpassing rates of other chronic conditions (11). It is estimated that 5-10% of individuals with pre-DM progress to DM annually, and over 70% eventually develop DM (6). Importantly, lifestyle modifications have been shown to reduce the risk of diabetes in prediabetic individuals by as much as 58% (12). Therefore, identifying risk factors for pre-DM and implementing targeted interventions are essential steps in preventing the onset of DM.

Recent studies have identified a strong association between high-density lipoprotein cholesterol (HDL-c), Gamma-glutamyl transferase (GGT), and metabolic disorders such as nonalcoholic fatty liver disease (NAFLD) and DM (13–17). Findings from two cross-sectional studies revealed that an increased GGT/HDL-c ratio (GHR) is significantly linked to both NAFLD and metabolically associated fatty liver disease (MAFLD) (18–20). Besides, evidence suggests that NAFLD is closely connected to glucose metabolism disorders, with GGT, HDL-c, and NAFLD all playing roles in the development of insulin resistance (IR) (21–24). Additional studies have demonstrated that DM and NAFLD share overlapping pathophysiological mechanisms, including inflammation, oxidative stress, and IR (25, 26). A study from China found that after adjusting for potential confounding factors, each 1-unit increase in the GHR is associated with a 1.3% increase in the incidence of DM (HR = 1.013, 95% CI: 1.002, 1.024) (27). Given that IR is the core pathophysiological mechanism underlying the onset and progression of DM, we hypothesize that an elevated GHR may be closely associated with pre-DM, which serves as the intermediate metabolic state in the transition from normoglycemia to DM. However, to date, no studies have systematically explored the relationship between the GHR and the risk of pre-DM. Therefore, this study aims to conduct a cohort study among Chinese adults with normoglycemia to investigate the association between the GHR and pre-DM, validate this hypothesis, and provide new scientific evidence for the early screening and intervention of pre-DM.

## Methods

### Study design and study population

**Figure 1:**
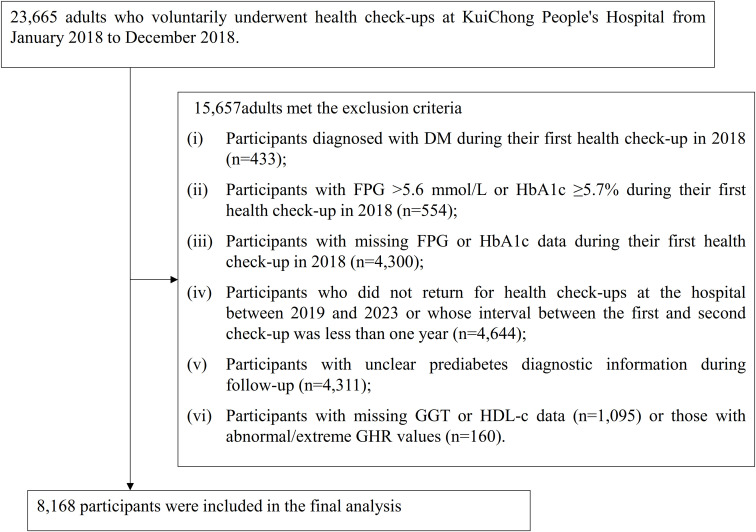
Flowchart of the study participant screening process.

This retrospective cohort study analyzed medical records of individuals who voluntarily participated in health examinations at Shenzhen Dapeng New District Kuichong People’s Hospital between January 2018 and December 2023. The baseline cohort consisted of 23,665 participants over the age of 20 who underwent health check-ups during the initial period from January to December 2018. The exclusion criteria were as follows: (i) Participants diagnosed with DM during their first health check-up in 2018 (n=433); (ii) Participants with FPG >5.6 mmol/L or HbA1c ≥5.7% during their first health check-up in 2018 (n=554); (iii) Participants with missing FPG or HbA1c data during their first health check-up in 2018 (n=4,300); (iv) Participants who did not return for health check-ups at the hospital between 2019 and 2023 or whose interval between the first and second check-up was less than one year (n=4,644); (v) Participants with unclear pre-DM diagnostic information during follow-up (n=4,311); (vi) Participants with missing GGT or HDL-c data (n=1,095) or participants with abnormal/extreme GHR values (n=160), where abnormal/extreme values are defined as those falling below or above three standard deviations from the mean (28, 29). Ultimately, 8,168 participants were included in the final analysis. **Figure 1** illustrates the process undertaken to screen and select the study participants.

### Ethical approval and consent

The Ethics Committee of Shenzhen Dapeng New District Kuichong People’s Hospital approved this study (Ethics Approval Number: 2024005). Given the retrospective design and the complete anonymization of all data, the committee granted a waiver for informed consent. Additionally, the study was conducted in full compliance with the ethical guidelines set forth in the Declaration of Helsinki, ensuring adherence to its principles and relevant ethical standards and regulations.

### Variables

#### The Ratio of GGT to HDL-c

The gamma-glutamyl transferase to high-density lipoprotein cholesterol ratio (GHR) was analyzed as a continuous variable. This ratio was calculated by dividing serum GGT (measured in U/L) by HDL-c (measured in mmol/L).

#### Definition of prediabetes

Pre-DM was identified based on the American Diabetes Association’s diagnostic criteria. Specifically, participants were classified as having pre-DM if they maintained normal blood glucose at baseline, did not progress to DM during follow-up, and exhibited either fasting plasma glucose (FPG) values ranging from 5.6 to <7.0 mmol/L or hemoglobin A1c (HbA1c) levels between 5.7% and <6.5% (30).

#### Covariates

The selection of covariates was based on previous studies and our clinical expertise (16, 31, 32). Variables used as covariates included: (i) Continuous variables: age, body mass index (BMI), alanine aminotransferase (ALT), aspartate aminotransferase (AST), systolic blood pressure (SBP), diastolic blood pressure (DBP), low-density lipoprotein cholesterol (LDL-c), total cholesterol (TC), triglycerides (TG), glycated hemoglobin (HbA1c), fasting plasma glucose (FPG), high-sensitivity C-reactive protein(Hs-CRP), and serum creatinine (Scr). (ii) Categorical variables: sex, hypertension, drinking status, physical activity, and smoking status.

#### Data collection

Qualified physical examination staff conducted physical examinations and gathered baseline data on lifestyle factors, including alcohol intake and smoking habits, as well as demographic information, such as age and sex, along with hypertension status, utilizing a standardized questionnaire. Blood pressure measurements were taken from participants in the morning while they were fasting, sitting quietly for 5 minutes, using a standard mercury sphygmomanometer. All participants in the examination were required to fast for at least 10 hours before the collection of fasting venous blood samples for comprehensive biochemical tests. Biochemical parameters, including FPG, TC, TG, HDL-c, and LDL-c, were analyzed using the Beckman 5800 automatic analyzer. Additionally, hemoglobin levels were assessed with the Mindray 5180 hematology analyzer.

#### Handling of missing data

Missing data is a common phenomenon in observational studies and is often unavoidable. In this study, missing data were observed for several variables, including hypertension (65, 0.80%), SBP (16, 0.20%), DBP (16, 0.20%), Scr (278, 3.40%), smoking status (474, 5.80%), BMI (133, 1.63%), drinking status (774, 9.48%), and physical activity (1073, 13.14%). To reduce bias caused by missing variables, multiple imputations (five times) were used to handle the missing data (33, 34). The imputation model applied linear regression with 10 iterations and included the following variables: age, sex, BMI, AST, ALT, SBP, DBP, TG, LDL-c, TC, smoking status, physical activity, hypertension, drinking status, FPG, HbA1c, Scr, and CRP. The missing data were analyzed under the assumption of missing at random (MAR) (34). It should be noted that five imputed datasets were generated, allowing for separate estimates, such as means, regression coefficients, and so forth, to be made for each dataset. Rubin’s Rules were then applied to combine these estimates.

#### Statistical analysis

Statistical analyses were conducted using R software version 3.4.3 and Empower(R) software version 4.2. A P-value of less than 0.05 (two-sided) was considered statistically significant. Participants were categorized based on quartiles of GHR. For continuous variables that followed a normal distribution, both the mean and standard deviation were reported. In contrast, for variables exhibiting a skewed distribution, the median and interquartile range were presented. Categorical variables were summarized using percentages and frequencies. To evaluate statistical significance across groups, the Kruskal-Wallis H test was applied for skewed variables, one-way analysis of variance (ANOVA) was utilized for normally distributed variables, and the chi-squared (χ²) test was employed for categorical variables.

Univariate and multivariate Cox proportional hazards regression models were used to explore the association between GHR and pre-DM risk. Three models were applied: Model I was unadjusted, Model II was minimally adjusted for sex and age, and Model III was fully adjusted for sex, BMI, age, drinking status, ALT, TG, FPG, DBP, smoking status, Scr, AST, hypertension, and SBP. TC was excluded from the multivariate analysis due to collinearity with other variables (**Supplementary Table S1**). Additionally, patients diagnosed with DM during follow-up could interfere with the assessment of normoglycemia progression to pre-DM. Therefore, a competing risk multivariate Cox proportional hazards regression, based on the Fine and Gray method, was used to verify the relationship between GHR and pre-DM, treating the progression from normoglycemia to DM as a competing event (35, 36).

Additionally, a Cox proportional hazards regression model incorporating a cubic spline function was employed to examine the potential non-linear association between the GHR and the risk of pre-DM. If nonlinearity was identified, a recursive algorithm was applied to pinpoint the inflection point. Following this, a two-part Cox proportional hazards regression model was developed for each side of the identified inflection point. Ultimately, the optimal Model that best described the relationship between GHR and pre-DM risk was selected based on the log-likelihood ratio test.

Previous research has demonstrated a significant link between hypertension, obesity, and glucose metabolism (37–39). To confirm the findings of our study, we performed several sensitivity analyses. Initially, we limited the analysis to participants with BMI<28 kg/m² (40). Additionally, we excluded individuals with hypertension from sensitivity analyses. Besides, a generalized additive model (GAM) was utilized to integrate continuous covariates into the multivariate Cox proportional hazards regression equation. Lastly, to evaluate the potential impact of unmeasured confounding variables on the relationship between the GHR and the risk of pre-DM, we calculated the E-value (41).

A stratified Cox proportional hazards regression model was applied for subgroup analyses based on factors such as drinking status, sex, smoking status, age, physical activity, SBP, and. Continuous variables, including age, SBP, and DBP, were categorized using clinical thresholds (age: <30, 30–40, 40–50, ≥50 years; DBP: <90, ≥90 mmHg; SBP: <140, ≥140 mmHg). Adjustments were made for covariates such as sex, BMI, age, drinking status, ALT, TG, FPG, DBP, smoking status, Scr, AST, hypertension, and SBP, in addition to the stratification factors. To assess potential interactions, the likelihood ratio test was conducted to compare models with and without interaction terms.

## Results

### Participant characteristics

**Figure 2:**
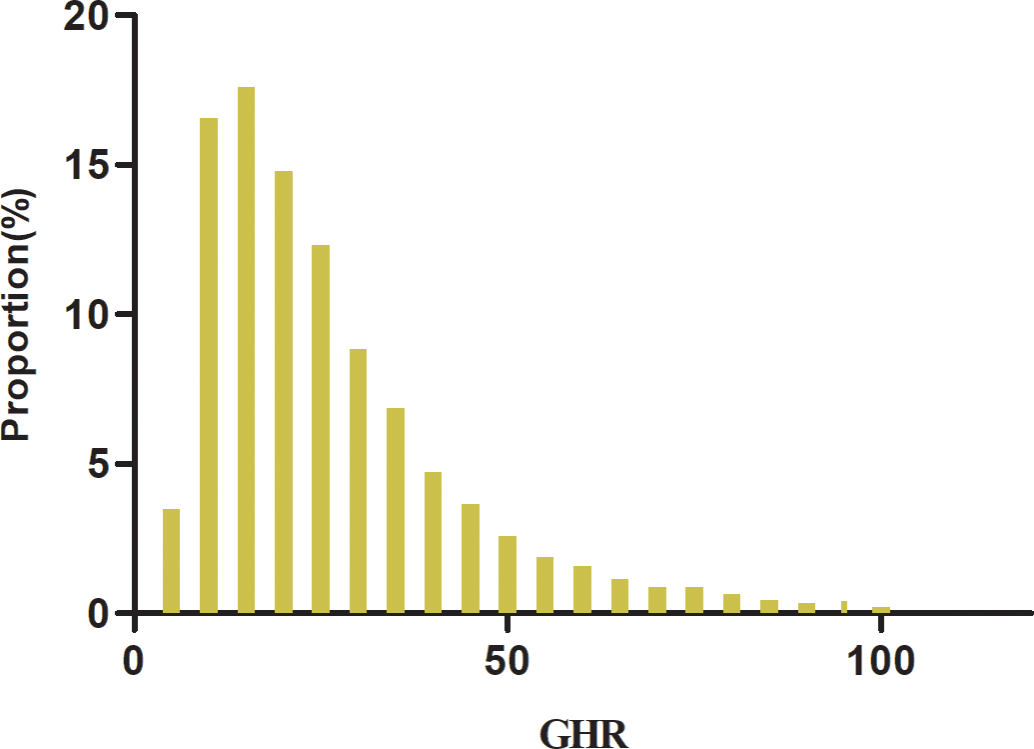
Distribution of GHR. **Figure 2** shows that the GHR had a left-skewed distribution ranging from 3.90 to 102.79 with a median value of 21.57 and an interquartile range (IQR) of 13.93 to 33.35.

A total of 8,168 participants are included in the study, consisting of 2,170 women and 5,998 men, with a mean age of 41.48 years (SD: 8.52). The GHR exhibits a left-skewed distribution, ranging from 3.90 to 102.79, with a median value of 21.57 and an interquartile range (IQR) of 13.93–33.35 (**Figure 2**). **Table 1** summarizes the anthropometric and biochemical characteristics of participants across GHR quartiles. The findings reveal that individuals in higher GHR quartiles tend to have elevated levels of LDL-c, age, DBP, SBP, BMI, ALT, TC, AST, TG, Scr, Hs-CRP, and HbA1c compared to those in the lowest quartile. Conversely, HDL-c levels are lower in participants with higher GHR values. Furthermore, the proportion of males, smokers, individuals engaging in low physical activity, and current drinkers is noticeably greater in the upper GHR quartiles compared to the lowest quartile.

**Table 1:** The baseline characteristics of participants with normoglycemia.

GHR quartiles	Q1 (<13.92)	Q2 (13.92-21.56)	Q3 (21.56-33.50)	Q4 (≥33.50)	P-value
N	2042	2038	2045	2043	
Age (years)	39.84 ± 8.37	41.49 ± 8.69	42.31 ± 8.61	42.28 ± 8.17	<0.001
SBP (mmHg)	110.07 ± 11.33	115.54 ± 10.80	118.83 ± 11.36	121.12 ± 12.61	<0.001
DBP (mmHg)	71.51 ± 7.49	75.16 ± 7.31	77.34 ± 7.28	78.94 ± 7.97	<0.001
BMI (kg/m^2^)	21.13 ± 3.02	22.82 ± 3.24	24.10 ± 3.43	25.43 ± 3.80	<0.001
TC (mmol/L)	2.12 ± 0.36	2.19 ± 0.41	2.24 ± 0.41	2.33 ± 0.43	<0.001
LDL-c (mmol/L)	1.22 ± 0.33	1.39 ± 0.37	1.44 ± 0.37	1.50 ± 0.39	<0.001
TG (mmol/L)	2.02 (1.60-2.72)	2.43 (1.89-3.26)	3.08 (2.28-4.06)	3.90 (2.84-5.30)	<0.001
HDL-c (mmol/L)	1.61 ± 0.34	1.31 ± 0.26	1.15 ± 0.24	1.05 ± 0.24	<0.001
FPG (mg/dL)	4.58 ± 0.38	4.74 ± 0.39	4.82 ± 0.38	4.88 ± 0.37	<0.001
AST (u/L)	24.68 ± 11.19	27.46 ± 11.26	29.45 ± 9.82	33.82 ± 10.70	<0.001
ALT (u/L)	26.89 ± 9.57	34.06 ± 12.15	40.32 ± 14.60	52.68 ± 23.19	<0.001
Hs-CRP (mg/L)	0.90 (0.40-2.10)	0.90 (0.50-2.00)	1.20 (0.60-2.20)	1.60 (0.80-3.00)	<0.001
GGT (u/L)	16.08 ± 3.85	22.81 ± 4.85	30.88 ± 7.15	53.02 ± 20.33	<0.001
Scr (umol/L)	69.07 ± 15.39	70.04 ± 15.24	69.92 ± 15.42	70.58 ± 15.82	0.019
HBA1C (%)	5.00 ± 0.24	5.10 ± 0.24	5.15 ± 0.24	5.19 ± 0.23	<0.001
Sex (n,%)					<0.001
Female	1394 (68.27%)	496 (24.34%)	184 (9.00%)	96 (4.70%)	
Male	648 (31.73%)	1542 (75.66%)	1861 (91.00%)	1947 (95.30%)	
Hypertension (n,%)	83 (4.06%)	148 (7.26%)	225 (11.00%)	298 (14.59%)	<0.001
Smoking (n,%)	124 (6.07%)	147 (7.21%)	157 (7.68%)	198 (9.69%)	<0.001
Physical Activity (n,%)					<0.001
Sedentary	370 (18.12%)	371 (18.20%)	432 (21.12%)	542 (26.53%)	
Light activity	768 (37.61%)	749 (36.75%)	786 (38.44%)	810 (39.65%)	
Moderate activity	697 (34.13%)	705 (34.59%)	665 (32.52%)	555 (27.17%)	
Vigorous activity	207 (10.14%)	213 (10.45%)	162 (7.92%)	136 (6.66%)	
Drinking status (n,%)					<0.001
Never	93 (4.55%)	33 (1.62%)	56 (2.74%)	53 (2.59%)	
Current	250 (12.24%)	306 (15.01%)	301 (14.72%)	314 (15.37%)	
Ever	1699 (83.20%)	1699 (83.37%)	1688 (82.54%)	1676 (82.04%)	

Continuous variables were summarized as mean (SD) or medians (quartile interval); categorical variables were displayed as percentage (%):

GGT, γ-glutamyl transferase; GHR, the ratio of γ-glutamyl transferase to high-density lipoprotein cholesterol; HbA1c, Hemoglobin A1c; LDL-c, low-density lipid cholesterol; FPG, fasting plasma glucose; SBP, systolic blood pressure; TG triglyceride; BMI, body mass index; Hs-CRP, high-sensitivity C-reactive protein; TC, total cholesterol, ALT, alanine aminotransferase; DBP, diastolic blood pressure; HDL-c, high-density lipoprotein cholesterol; AST aspartate aminotransferase; Scr, serum creatinine.

#### The incidence of prediabetes

**Figure 3:**
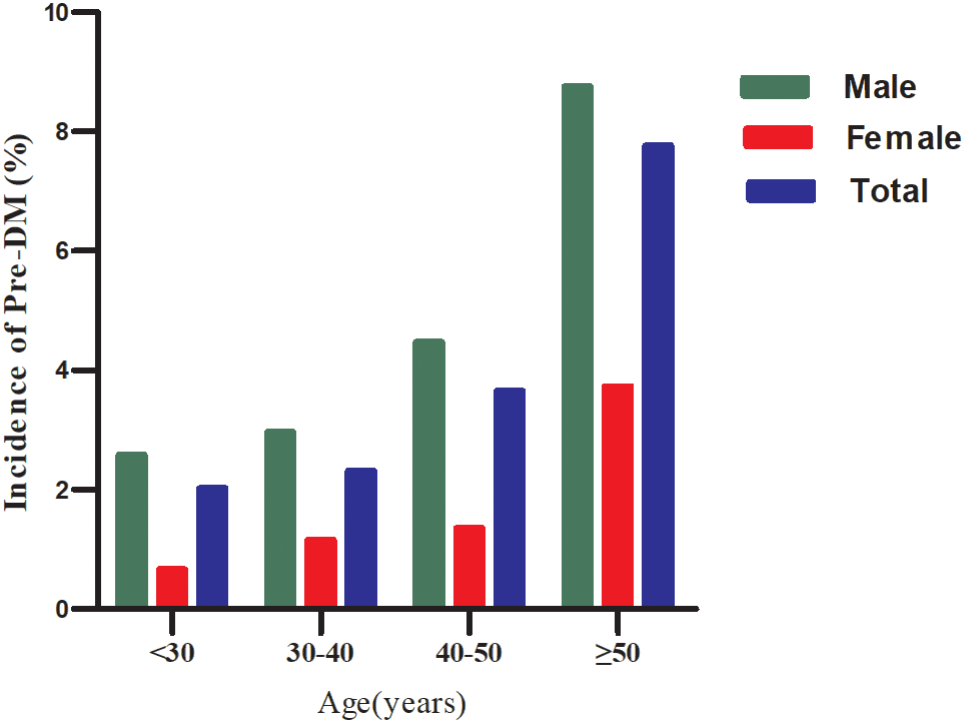
Incidence of prediabetes stratified by age in 10 intervals and sex.

During a median follow-up of 1.96 years, 311 participants (10.63%) transition from normoglycemia to pre-DM. The incidence rates of pre-DM across the GHR quartiles are 95.52, 194.24, 332.84, and 505.01 cases per 10,000 person-years, respectively. The cumulative incidence of pre-DM is 2.81%, with a quartile-specific cumulative incidence of 1.27% in Q1, 2.65% in Q2, 4.4% in Q3, and 6.9% in Q4. Participants in the highest GHR quartile (Q4) exhibit a significantly greater risk of developing pre-DM compared to those in the lowest quartile (Q1) (p<0.001 for trend) (**Table 2**). When stratified by 10-year age intervals, males consistently show a higher likelihood of progressing to pre-DM than females, regardless of age group. Moreover, the incidence of pre-DM increases with advancing age in both sexes (**Figure 3**).

**Table 2:** Incidence of prediabetes (% or per 10,000 person-years).

GHR	Participants (n)	Pre-DM events (n)	Incidence rate (95% CI) (%)	Per 10000 person-year
Total	6225	311	3.81 (3.39-4.22)	279.95
Q1 (<13.92)	2042	26	1.27 (0.79-1.76)	95.52
Q2 (13.92-21.56)	2038	54	2.65 (1.96-3.35)	194.24
Q3 (21.56-33.50)	2045	90	4.40 (3.51-5.29)	332.84
Q4 (≥33.50)	2043	141	6.90 (5.80-8.0)	505.01
P for trend			<0.001	

GHR, the ratio of γ-glutamyl transferase to high-density lipoprotein cholesterol; Pre-DM, prediabetes.

#### The influencing factors of prediabetes were analyzed using univariate Cox proportional hazards regression

**Figure 4:**
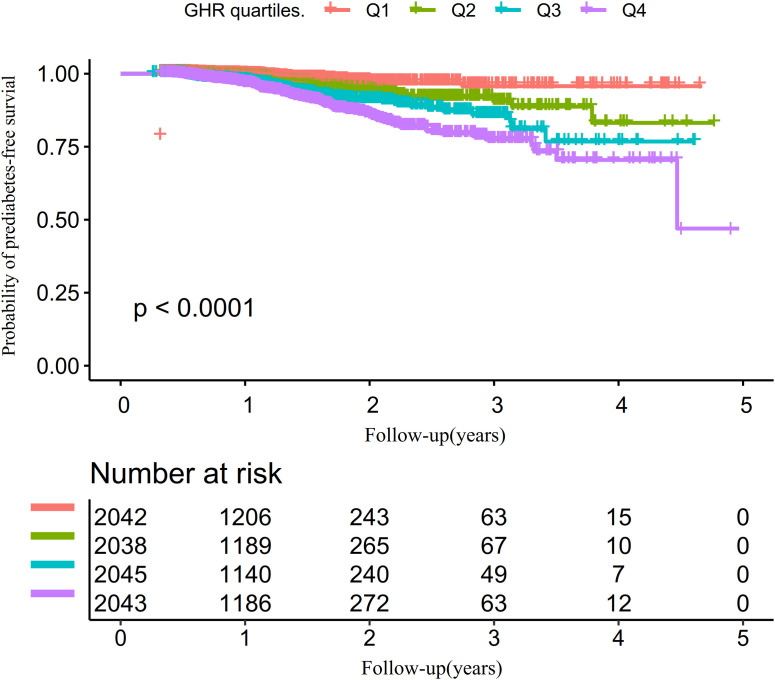
Kaplan–Meier event-free survival curve.

The univariate analysis revealed that the risk of progression from normoglycemia to pre-DM was positively correlated with factors such as age, male sex, hypertension, SBP, BMI, DBP, AST, TG, ALT, FPG, GGT, BUN, and current alcohol consumption (all P<0.05). In contrast, HDL-c exhibited a negative association with pre-DM risk. No significant relationships were identified between pre-DM and smoking, Scr, LDL-c, or Hs-CRP (all P>0.05) (**Supplementary Table S2**). Kaplan-Meier survival curves, stratified by GHR quartiles and shown in **Figure 4**, illustrate the probability of prediabetes-free survival differed significantly between the GHR quartiles (log-rank test, p<0.001). The probability of prediabetes-free survival gradually decreased with increasing GHR, suggesting that participants with the highest GHR had the greatest risk of pre-DM.

#### The relationship between GHR and the risk of progression from normoglycemia to prediabetes

To investigate the association between GHR and the risk of pre-DM, we established three Cox proportional hazards regression models. In Model I, each 5-unit increase in GHR was linked to an 11.2% higher risk of progression from normoglycemia to pre-DM (HR = 1.112, 95% CI: 1.085–1.139). Model II, adjusted for demographic variables, demonstrated that a 5-unit rise in GHR was significantly associated with a 10.2% increase in pre-DM risk (HR = 1.102, 95% CI: 1.074–1.131). Finally, Model III, which incorporated adjustments for a range of potential confounders, further validated the relationship, showing that each 5-unit increment in GHR corresponded to a 6.1% increase in the risk of pre-DM (HR = 1.061, 95% CI: 1.028–1.095) (**Table 3**).

**Table 3:** The relationship between GHR and the risk of progression from normoglycemia to prediabetes.

Exposure	Pre-DM events (n)	Model I (HR,95%CI) P	Model II (HR,95%CI) P	Model III (HR,95%CI) P	Model IV (HR,95%CI) P
GHR (per 5-unit)	311	1.112 (1.085, 1.139) <0.001	1.102 (1.074, 1.131) <0.001	1.061 (1.028, 1.095) <0.001	1.044 (1.009, 1.080) 0.013
GHR5 quartile					
Q1	26	Ref	Ref	Ref	Ref
Q2	54	2.066 (1.294, 3.298) 0.002	1.784 (1.112, 2.861) 0.016	1.518 (0.941, 2.449) 0.087	1.203 (0.736, 1.968) 0.461
Q3	90	3.696 (2.389, 5.719) <0.001	2.912 (1.867, 4.542) <0.001	2.233 (1.411, 3.532) <0.001	1.653 (1.018, 2.684) 0.042
Q4	141	5.359 (3.527, 8.143) <0.001	4.305 (2.802, 6.614) <0.001	2.676 (1.674, 4.278) <0.001	1.865 (1.132, 3.073) 0.014
P for trend		<0.001	<0.001	<0.001	0.003

Model I: we did not adjust other covariates.

Model II: we adjust sex, age.

Model III: we adjust sex, BMI, age, drinking status, ALT, TG, FPG, DBP, physical activity, smoking status, Scr, AST, hypertension, and SBP.

Model IV: we adjusted sex, BMI (smooth), age (smooth), drinking status, ALT (smooth), physical activity, TG (smooth), AST (smooth), DBP (smooth), Scr, FPG, (smooth), smoking, and drinking status, SBP (smooth).

HR, hazard ratio; Ref, reference; CI, confidence.

Additionally, GHR was transformed from a continuous variable into a categorical one and reintroduced into the Cox proportional hazards regression model. In the multivariable-adjusted Model, using the first quartile (Q1, 26 pre-DM events) of GHR as the reference group, HRs for progression to pre-DM were 1.518 (95% CI: 0.941–2.449) for the second quartile (Q2, 54 pre-DM events), 2.233 (95% CI: 1.411–3.532) for the third quartile (Q3, 90 pre-DM events), and 2.676 (95% CI: 1.674–4.278) for the fourth quartile (Q4, 141 pre-DM events). This indicates that, compared to individuals in Q1, those in Q2 had a 51.8% higher risk of progression from normoglycemia to pre-DM, while participants in Q3 and Q4 had a 123.3% and 167.6% increased risk, respectively (**Table 3** - Model III).

#### The competitive risk multivariate Cox proportional hazards regression results

**Table 4** presented the findings of the competing risk analysis, which accounted for the progression from normoglycemia to DM as a competing event for the progression from normoglycemia to pre-DM. In Model I, a positive association was observed between GHR and the risk of pre-DM, with a subdistribution hazard ratio (SHR) of 1.11 (95% CI: 1.09–1.14) per 5-unit increase in GHR. After adjusting for sex and age in Model II, the SHR for the association between GHR and pre-DM risk was 1.09 (95% CI: 1.06–1.12) per 5-unit increment. In the fully adjusted Model (Model III), which controlled for potential confounders such as sex, BMI, age, drinking status, ALT, TG, FPG, DBP, smoking, Scr, AST, hypertension, and SBP, the positive relationship between GHR(per 5-unit) and pre-DM risk persisted, with an SHR of 1.05 (95% CI: 1.02–1.09).

**Table 4:** The relationship between GHR and the progression from normoglycemia to pre-DM in different competing risk models.

Exposure	Pre-DM events(n)	Model (SHR,95%CI) P	Model II (SHR,95%CI), P	Model III (SHR,95%CI) P
GHR (per 5-unit)	311	1.11 (1.09, 1.14) <0.001	1.09 (1.06, 1.12)	1.05 (1.02, 1.09) <0.001
GHR5 quartile				
Q1	26	Ref.	Ref.	Ref.
Q2	54	2.07 (1.29, 3.30) <0.001	1.51 (0.92, 2.49) 0.1030	1.37 (0.83, 2.26) <0.001
Q3	90	3.70 (2.39, 5.72) <0.001	2.39 (1.48, 3.87) <0.001	1.96 (1.20, 3.21) <0.001
Q4	141	5.36 (3.53, 8.14) <0.001	3.51 (2.19, 5.62) <0.001	2.35 (1.42, 3.88) <0.001
P for trend		<0.001	<0.001	<0.001

Model I: we did not adjust other covariates.

Model II: we adjust sex, age.

Model III: we adjust sex, BMI, age, drinking status, ALT, TG, FPG, physical activity, DBP, smoking status, Scr, AST, hypertension, and SBP.

The same pattern was observed when GHR was treated as a categorical variable. In the fully adjusted Model, participants in the second quartile(Q2, 54 pre-DM events) exhibited a 37% higher risk of developing pre-DM compared to those in the first quartile (Q1, 26 pre-DM events) (SHR = 1.37, 95% CI: 0.83–2.26). Those in the third quartile (Q3, 90 pre-DM events) had a 96% increased risk (SHR = 1.96, 95% CI: 1.20–3.21), while participants in the fourth quartile(Q4, 141 pre-DM events) demonstrated a 135% higher risk (SHR = 2.35, 95% CI: 1.42–3.88) relative to Q1.

#### Sensitivity analysis

To validate the robustness of our findings, we conducted multiple sensitivity analyses. First, continuous covariates were incorporated into the Model as smooth curves using GAM. As shown in **Table 3** (Model IV), the results were largely consistent with those of the fully adjusted Model. Specifically, the analysis indicated that GHR (per 5-unit increase) was associated with an elevated risk of progressing from normoglycemia to pre-DM, with an HR of 1.044 (95% CI: 1.009–1.080). A sensitivity analysis was also performed on a subset of 6,205 participants with a BMI below 28 kg/m². After adjusting for potential confounders, the positive association between GHR (per 5-unit increase) and pre-DM risk persisted (HR = 1.062, 95% CI: 1.025–1.100). Furthermore, when individuals with hypertension were excluded from the analysis(n=7,441), the relationship between GHR (per 5-unit increase) and pre-DM risk remained significant after adjusting for confounding factors (HR = 1.059, 95% CI: 1.012–1.109) (**Table 5**).

**Table 5:** The relationship between GHR and the progression from normoglycemia to pre-DM in different sensitivity analyses.

Exposure		Model I(HR,95%CI) P		Model II (HR,95%CI) P
	Pre-DM events(n)	HR(95%CI) P	Pre-DM events(n)	HR(95%CI) P
GHR (per 5-unit)	236	1.062 (1.025, 1.100) <0.001	245	1.059 (1.012, 1.109) 0.014
GHR quartiles				
Q1	25	Ref	25	Ref
Q2	48	1.456 (0.889, 2.385) 0.135	48	1.346 (0.797, 2.274) 0.266
Q3	77	1.895 (1.171, 3.067) 0.009	68	2.005 (1.206, 3.331) 0.007
Q4	86	2.320 (1.416, 3.803) <0.001	104	2.145 (1.242, 3.704) 0.006
P for trend		<0.001		0.002

Model I involved a sensitivity analysis of participants with BMI <28 kg/m^2^ (n=6,205). sex, age, drinking status, ALT, TG, FPG, physical activity, DBP, smoking status, Scr, AST, hypertension, and SBP were adjusted.

Model II involved sensitivity analyses after excluding participants with hypertension (N=7,414). sex, BMI, age, drinking status, ALT, TG, FPG, physical activity, DBP, smoking status, Scr, AST, and SBP were adjusted.

In addition, we observe that the E-value is 1.32, which exceeds the relative risk of 1.27 for the relationship between unmeasured confounders and GHR and is lower than the relative risk of 1.37 for the relationship between unmeasured confounders and pre-DM. This suggests that unknown or unmeasured confounders are unlikely to have a significant impact on the relationship between GHR and the risk of progression from normoglycemia to pre-DM. Furthermore, to evaluate the validity of the multiple imputation data, we compared the datasets obtained before and after the imputation. The results showed no significant differences in baseline characteristics between the two groups, with a p-value greater than 0.05 for the inter-group comparison. This indicates that the data are consistent, enhancing the credibility and validity of analyses conducted using the multiple imputation data (**Supplementary Table S3**). These comprehensive sensitivity analyses support the robustness and reliability of our research findings.

#### Non-linear relationship between GHR and the risk of progression from normoglycemia to pre-DM

**Figure 5:**
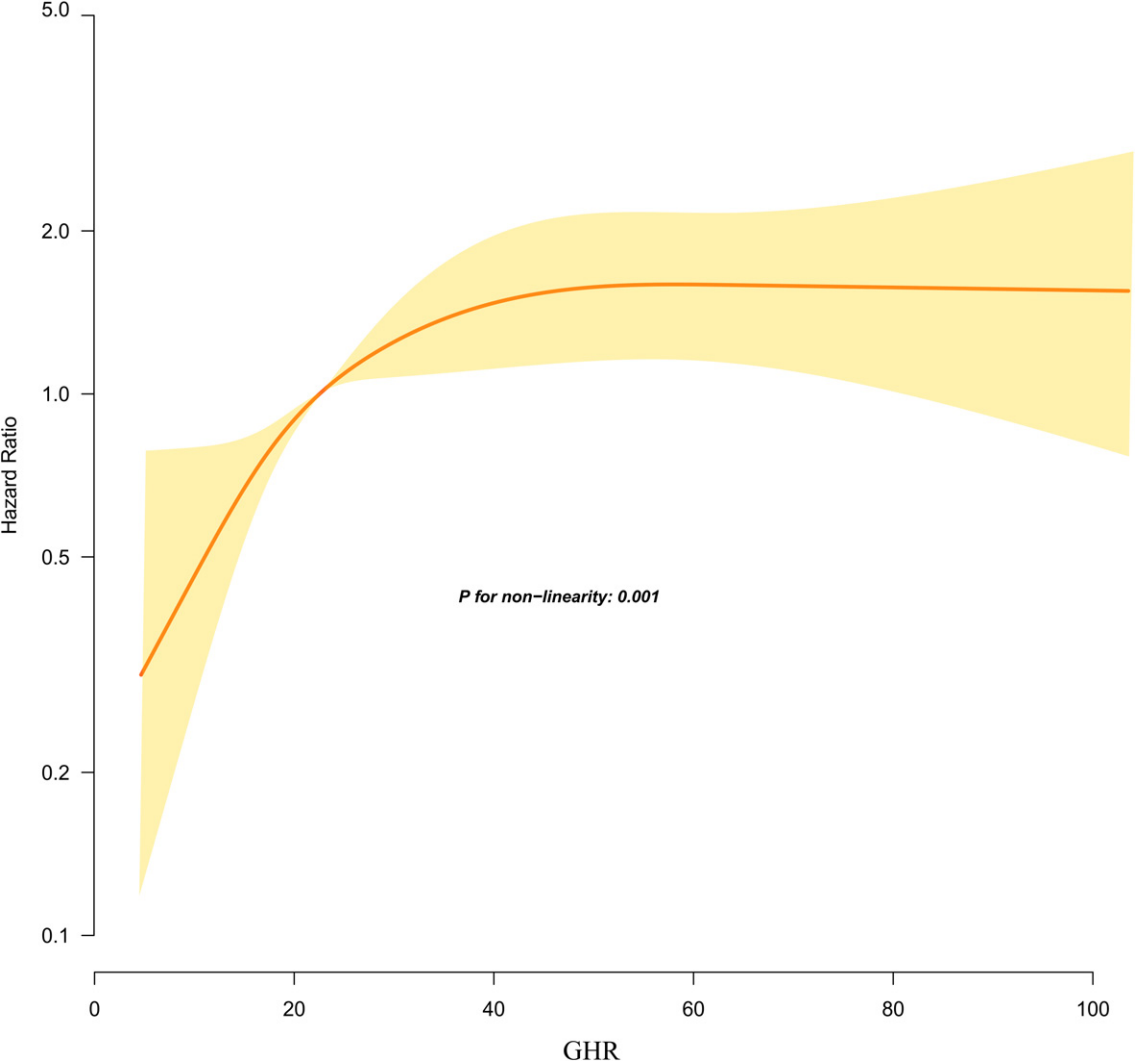
Kaplan–Meier event-free survival curve. The probability of prediabetes-free survival differed significantly between the GHR quartiles (log-rank test, p<0.001). The probability of prediabetes-free survival gradually decreased with increasing GHR, suggesting that the group with the highest GHR had the highest risk of prediabetes. Non-linear relationship between GHR and the risk of progression from normoglycemia to prediabetes.

Using the Cox proportional hazards regression model with cubic spline functions, we identify a non-linear association between GHR and the risk of progression from normoglycemia to pre-DM (**Figure 5**). Through a recursive algorithm, the inflection point for GHR is determined to be 24.37. To further explore this relationship, a two-segment Cox proportional hazards regression model is applied to estimate the HRs on either side of the inflection point. Below the inflection point, each 5-unit increase in GHR is associated with a significantly higher risk of progression to pre-DM (HR = 1.394, 95% CI: 1.197–1.623). However, above the inflection point, the HR is 1.024 (95% CI: 0.985–1.065), which is not statistically significant (**Table 6**).

**Table 6:** The result of two-piecewise linear regression model.

Outcome:	HR (95%CI)	p-value
Fitting Model by two-piecewise Cox regression		
Inflection points of GHR	24.37	
<24.37(per 5-unit)	1.394 (1.197, 1.623)	<0.001
> 24.37(per 5-unit)	1.024 (0.985, 1.065)	0.223
P for log-likelihood ratio test	<0.001	

Adjusted variables included sex, BMI, age, drinking status, ALT, TG, FPG, physical activity, DBP, smoking status, Scr, AST, hypertension, and SBP.

#### Subgroup analysis

Across all predefined and exploratory subgroup analyses (**Supplementary Table S4**), no significant interactions were observed between GHR and variables such as sex, age, smoking, SBP, DBP, physical activity, or drinking status (P > 0.05 for interactions). These findings suggest that none of these factors significantly influence or modify the association between GHR and the risk of progression from normoglycemia to pre-DM.

## Discussion

This retrospective cohort study revealed an independent positive link between GHR and the risk of progression from normoglycemia to pre-DM. Moreover, a saturation effect curve was identified, with an inflection point at a GHR value of 24.37. The association between GHR and the risk of progression to pre-DM varied on either side of this threshold.

The rising global prevalence of pre-DM has become a significant public health concern (42). The term “pre-DM” is used to identify individuals who are at risk of developing DM in the future. However, pre-DM is also associated with a high burden of cardiometabolic risk factors, DM-related complications, and adverse outcomes (6, 8, 43, 44). For example, one study suggests that abnormal glucose metabolism can induce a series of systemic metabolic and non-metabolic abnormalities, potentially leading to myocardial dysfunction, such as inflammation, fibrosis, and myocardial stiffening, which may result in heart failure with preserved ejection fractions (44). Therefore, identifying the risk factors for pre-DM and implementing early interventions are essential for preventing DM and its complications.

GGT, a biomarker commonly used to evaluate hepatocellular damage, is widely recognized as a predictor of NAFLD. Previous studies have demonstrated a significant association between elevated GGT levels and IR (45–47). Moreover, research has established a positive link between GGT and glucose metabolism disorders. For instance, a cohort study conducted in Korea involving 4,088 men reported that, compared to individuals with GGT levels below 9 U/L (31% of participants), the adjusted relative risks (RR) for DM incidence were 8.0, 13.3, 12.6, 19.6, and 25.8 for those with GGT concentrations of 10–19, 20–29, 30–39, 40–49, and above 50 U/L, respectively (48). Similarly, another Korean cohort study found that, after adjusting for confounding factors, men in the highest quartile of serum GGT levels had a 2.55-fold increased risk of developing DM compared to those in the lowest quartile (HR = 2.55, 95% CI: 1.86–3.51). Meanwhile, women in the highest quartile exhibited a 90% higher risk (HR = 1.90, 95% CI: 1.40–2.58) (49). Further supporting these findings, a cohort study of Japanese men observed comparable results. After adjusting for potential confounders, participants in the fifth quintile of GGT levels had a 1.44-fold increased risk of DM compared to those in the first quintile (RR = 2.44, 95% CI: 1.34–4.46) (50). In addition to GGT, HDL-c levels have been shown to have a significant inverse relationship with IR (51, 52). A cohort study in the United States involving 2,829 participants found that higher HDL-c levels were associated with a reduced risk of DM, with an HR of 0.78 (95% CI: 0.50–0.63) per standard deviation increase (53). Similarly, a secondary analysis of data from a Japanese cohort study revealed that low HDL-c levels were linked to an elevated risk of DM (HR = 0.54, 95% CI: 0.35–0.82 (54). Likewise, a study focusing on middle-aged and elderly individuals in China reported similar conclusions. Compared to participants with HDL-c levels below 1.15 mmol/L, the adjusted HRs for those with HDL-c levels of 1.15–1.39 mmol/L, 1.40–1.69 mmol/L, and ≥1.70 mmol/L were 0.98 (95% CI: 0.62–1.55), 0.48 (95% CI: 0.27–0.85), and 0.44 (95% CI: 0.25–0.80), respectively (55). Based on these findings, it can be inferred that GHR may be positively associated with the risk of progression from normoglycemia to pre-DM. Unfortunately, no studies to date have explored this potential relationship. Our findings provide evidence supporting this hypothesis. Furthermore, we analyzed this relationship by examining GHR as both a continuous and categorical variable, which allowed us to minimize information loss and quantify the association more precisely. Sensitivity analyses focusing on participants with BMI below 28 kg/m² and those without hypertension further validated the consistency of these findings within specific subgroups. Additionally, the results of the competing risk multivariate Cox proportional hazards regression analysis were consistent with those obtained from the standard multivariate Cox proportional hazards model, reinforcing the robustness of our conclusions. In conclusion, elucidating the relationship between GHR and pre-DM holds significant clinical value. Incorporating GHR into routine clinical assessments could enable healthcare providers to identify high-risk individuals at an earlier stage, facilitating timely lifestyle or pharmacological interventions to prevent or delay the onset of pre-DM and ultimately reduce the incidence of DM.

Moreover, a nonlinear relationship between GHR and the risk of pre-DM was identified for the first time. The inflection point for GHR was determined to be 24.37. Below this threshold, each 5-unit increase in GHR was associated with a 39.4% higher risk of progressing from normoglycemia to pre-DM. However, when GHR exceeded 24.37, the relationship was no longer statistically significant. In other words, the risk of progression from normoglycemia to pre-DM increased significantly with higher GHR levels in patients. However, when GHR reached 24.37, further increases in GHR did not lead to additional increases in the risk of progression from normoglycemia to pre-DM. The study found that participants with GHR ≥24.37 had higher levels of age, SBP, DBP, LDL-c, TG, SBP, and ALT compared to those with GHR <24.37. Additionally, participants with GHR ≥24.37 had higher proportions of physical inactivity, hypertension, and smoking (**Supplementary Table S5**). However, these indicators are closely related to glucose metabolism and IR (56–59). When GHR exceeded 24.37, the impact of GHR on pre-DM became relatively weaker due to the presence of these risk factors. Conversely, in populations with GHR less than 24.37, these risk factors for diabetic metabolic disorders were at lower levels, resulting in a reduced impact on DM and a relatively stronger effect of GHR. This might explain the nonlinear relationship between GHR and pre-DM risk. The identification of this nonlinear association carries important clinical implications. It offers valuable guidance for clinical counseling and provides a reference for optimizing strategies to prevent pre-DM and DM. Specifically, maintaining GHR levels below 24.37 through dietary interventions and lifestyle modifications and further reducing GHR levels could significantly decrease the risk of progressing from normoglycemia to pre-DM.

This study presents several significant strengths: (i) It is the first to explore the association between GHR and the risk of pre-DM in individuals with normoglycemia; (ii) The study elucidated the nonlinear relationship between GHR and the risk of progression from normoglycemia to pre-DM and identified the inflection point. This represents a significant advance; (iii) multiple imputation techniques were utilized to address missing data, thereby enhancing statistical power and reducing bias associated with absent covariate information; and (iv) to validate our findings, we conducted a series of sensitivity analyses. These included transforming GHR into a categorical variable, incorporating continuous covariates as curves in GAM, utilizing competing risk models, and re-evaluating the relationship between GHR and pre-DM incidence after excluding participants with a BMI greater than 28 kg/m² or those with hypertension.

However, several limitations should be noted: First, the association between GHR and the progression from normoglycemia to pre-DM may differ among various ethnic groups, indicating that our results require further validation across diverse racial populations. We intend to collaborate with international researchers to explore these associations in cohorts with different genetic backgrounds. Second, as this study is a retrospective cohort study, we cannot adjust for unobserved factors. For example, this study did not collect information on hepatitis C viruses, which previous study has shown to have a significant association with DM and IR (60). To address this, we calculated E-values to evaluate the potential influence of unmeasured confounding factors, which indicated that such factors were unlikely to significantly impact our findings. In future investigations, we will include as many relevant variables as possible, including lifestyle factors, lipid-lowering medications, and information on hepatitis C virus infection, in order to conduct a more comprehensive analysis of the relationship between GHR and the progression from normoglycemia to pre-DM. Additionally, this study only measured GHR and other parameters at baseline, without assessing changes in GHR over time. Future research efforts will involve either conducting new studies or collaborating with other researchers to gather more comprehensive data, including longitudinal changes in GHR levels. Lastly, it is important to note that while this retrospective observational study can establish an independent association between GHR and the incidence of prediabetes, it cannot determine a causal relationship between the two. This needs to be further explored in future prospective studies.

## Conclusion

This study reveals a positive, nonlinear association between GHR and the risk of progression from normoglycemia to pre-DM in Chinese adults. Specifically, when GHR is below 24.37, a significant positive association exists between GHR and the risk of pre-DM. Healthcare providers and patients can work together to lower GHR levels through dietary interventions and lifestyle changes. Lowering GHR to at least below 24.37 and further decreasing it has the potential to substantially reduce the risk of progression from normoglycemia to pre-DM. This study provides valuable insights to support clinical consultations and optimize prevention strategies for pre-DM and DM.

## Data Availability

The raw data supporting the conclusions of this article will be made available by the authors, without undue reservation.
