# Supplementary material for: Nonlinear association between gamma-glutamyl transferase to high-density lipoprotein cholesterol ratio and risk of progression from normoglycemia to prediabetes: a 5-year cohort study
Source: Front Endocrinol (Lausanne). 2025 Jul 7;16:1552044. doi: 10.3389/fendo.2025.1552044 (PMC12277162; doi:10.3389/fendo.2025.1552044)
Supplement: Supplementary file 1 [file Table1.docx]

**Nonlinear Association Between Gamma-Glutamyl Transferase to High-Density Lipoprotein Cholesterol Ratio and Risk of Progression from** **Normoglycemia to Prediabetes：a 5-year cohort study.**

**Running title: GHR and prediabetes**

Chuang Gao^1#^,Cailing Yu^1#^, Peijie Shi ^1^^#^, Dehong Liu^2*^, Qiming Li^2*^, Yong Han^2*^

^1^ Department of Emergency, Shenzhen Dapeng New District Kuichong People's Hospital, Shenzhen 518000.

^2^ Department of Emergency, Shenzhen Second People's Hospital, Shenzhen 518035, Guangdong Province, China

Chuang Gao^1#^,Cailing Yu^1#^, Peijie Shi ^1#^ have contributed equally to this work.

*Corresponding author

Dehong Liu

Department of Emergency, Shenzhen Second People's Hospital

No.3002 Sungang Road, Futian District,

Shenzhen 518035,

Guangdong Province,

China

E-mail: dhliu_emergency@163.com

*Corresponding author

Qiming Li

Department of Emergency, Shenzhen Second People's Hospital

No.3002 Sungang Road, Futian District,

Shenzhen 518035,

Guangdong Province,

China.

[liqiming231@hotmail.com](mailto:liqiming231@hotmail.com)

***Corresponding author**

Yong Han

Department of Emergency,

Shenzhen Second People’s Hospital,

No.3002 Sungang Road, Futian District,

Shenzhen 518000,

Guangdong Province,

China

Hanyong511023@163.com

Table S1 Collinearity screening

|  | Step 1 | Step 2 |
| --- | --- | --- |
| GHR | 1.8 | 1.8 |
| Age | 1.2 | 1.2 |
| Sex | 1.7 | 1.5 |
| Hypertension | 4.4 | 4.4 |
| SBP | 3.1 | 3.1 |
| DBP | 3.1 | 3.1 |
| BMI | 1.5 | 1.5 |
| Smoking | 1 | 1 |
| TC | 10.1 | NA |
| LDL-c | 9.6 | 1.1 |
| TG | 2 | 1.2 |
| FPG | 1.2 | 1.2 |
| ALT | 1.7 | 1.7 |
| AST | 2.2 | 2.2 |
| CRP | 1 | 1 |
| physical activity | 1.1 | 1.1 |
| Scr | 1 | 1 |
| Drinking | 1 | 1 |

Variables excluded by collinearity screening: TC

Table S2. Factors affecting progression from normoglycemia to prediabetes analyzed by univariate Cox proportional hazards regression.

|  | Statistics | HR(95%CI) |
| --- | --- | --- |
| Age(years) | 41.480 ± 8.522 | 1.069 (1.057, 1.080) <0.001 |
| Sex |  |  |
| Female | 2170 (26.567%) | Ref |
| Male | 5998 (73.433%) | 3.291 (2.258, 4.795) <0.001 |
| Hypertension |  |  |
| No | 7414 (90.769%) | Ref |
| Yes | 754 (9.231%) | 3.308 (2.520, 4.343) <0.001 |
| SBP (mmHg) | 116.392 ± 12.268 | 1.034 (1.027, 1.041) <0.001 |
| DBP (mmHg) | 75.740 ± 8.015 | 1.057 (1.045, 1.069) <0.001 |
| BMI (kg/m^2^) | 25.871 ± 3.738 | 1.141 (1.116, 1.166) <0.001 |
| Smoking |  |  |
| No | 7542 (92.336%) | 1.0 |
| Yes | 626 (7.664%) | 1.375 (0.963, 1.964) 0.080 |
| TC (mg/dL) | 196.760 ± 36.470 | 1.003 (1.000, 1.006) 0.051 |
| LDL-c(mg/dL) | 122.793 ± 33.592 | 1.002 (0.999, 1.006) 0.145 |
| HDL-c(mg/dL) | 49.463 ± 13.323 | 0.969 (0.959, 0.979) <0.001 |
| TG (mg/dL) | 123.563 ± 77.426 | 1.001 (1.001, 1.002) <0.001 |
| FPG (mmol/L) | 4.755 ± 0.398 | 3.856 (2.471, 5.552) <0.001 |
| AST(u/L) | 28.854 ± 11.260 | 1.008 (1.002, 1.013) 0.007 |
| ALT(u/L) | 38.492 ± 18.367 | 1.012 (1.008, 1.015) <0.001 |
| CRP (mg/dL) | 2.283 ± 4.786 | 1.007 (0.990, 1.024) 0.426 |
| GGT(u/L) | 30.704 ± 17.869 | 1.016 (1.012, 1.021) <0.001 |
| Physical Activity |  |  |
| **Sedentary** | 1715 (20.997%) | Ref |
| **Light activity** | 3113 (38.112%) | 0.867 (0.646, 1.164) 0.341 |
| Moderate activity | 2622 (32.101%) | 0.961 (0.712, 1.295) 0.792 |
| Vigorous activity | 718 (8.790%) | 0.719 (0.438, 1.179) 0.191 |
| Scr(umol/L) | 69.905 ± 15.474 | 1.000 (0.993, 1.007) 0.940 |
| Drinking status |  |  |
| Never | 235 (2.877%) | Ref |
| current | 1171 (14.336%) | 1.968 (1.446, 4.579) 0.006 |
| ever | 6762 (82.786%) | 1.646 (0.733, 3.700) 0.228 |
| GHR | 5.267 ± 3.477 | 1.112 (1.085, 1.139) <0.001 |

Abbreviations: GGT, γ-glutamyl transferase; GHR, the ratio of γ-glutamyl transferase to high-density lipoprotein cholesterol; HbA1c,**Hemoglobin A1c;** LDL-c, low-density lipid cholesterol; FPG, fasting plasma glucose; SBP, systolic blood pressure; TG triglyceride; BMI, body mass index; TC, total cholesterol, ALT, alanine aminotransferase; DBP, diastolic blood pressure; HDL-c, high-density lipoprotein cholesterol; AST aspartate aminotransferase; Scr, serum creatinine.

Table S3 Comparison of baseline characteristics before and after multiple imputations.

| Characteristic | **Before multiple imputation** | **After multiple imputation** | P-value |
| --- | --- | --- | --- |
| N | 8168 | 8168 |  |
| Age(years) | 41.48 ± 8.52 | 41.48 ± 8.52 | 1.000 |
| SBP (mmHg) | 116.39 ± 12.26 | 116.39 ± 12.27 | 0.998 |
| DBP (mmHg) | 75.74 ± 8.02 | 75.74 ± 8.01 | 0.967 |
| BMI (kg/m^2^) | 25.86 ± 3.74 | 25.87 ± 3.74 | 0.949 |
| TC (mmol/l) | 5.09 ± 0.94 | 5.09 ± 0.94 | 1.000 |
| LDL-c (mmol/l) | 3.18 ± 0.87 | 3.18 ± 0.87 | 1.000 |
| TG (mmol/l) | 1.40 ± 0.87 | 1.40 ± 0.87 | 1.000 |
| HDL-c (mmol/l) | 1.28 ± 0.34 | 1.28 ± 0.34 | 1.000 |
| FPG (mmol/l) | 4.76 ± 0.40 | 4.76 ± 0.40 | 1.000 |
| AST(u/L) | 28.85 ± 11.26 | 28.85 ± 11.26 | 1.000 |
| ALT(u/L) | 38.49 ± 18.37 | 38.49 ± 18.37 | 1.000 |
| Hs-CRP (mg/dL) | 2.28 ± 4.79 | 2.28 ± 4.79 | 1.000 |
| GGT(u/L) | 30.70 ± 17.87 | 30.70 ± 17.87 | 1.000 |
| Scr(umol/L) | 69.92 ± 15.51 | 69.90 ± 15.47 | 0.953 |
| Sex |  |  | 1.000 |
| Female | 2170 (26.57%) | 2170 (26.57%) |  |
| Male | 5998 (73.43%) | 5998 (73.43%) |  |
| Hypertension | 724 (8.93%) | 754 (9.23%) | 0.992 |
| Physical Activity |  |  | 0.543 |
| **Sedentary** | 1315 (18.83%) | 1715 (21.00%) |  |
| **Light activity** | 2613(36.83%) | 3113 (38.11%) |  |
| Moderate activity | 2322 (32.73%) | 2622 (32.10%) |  |
| Vigorous activity | 845 (11.91%) | 718 (8.79%) |  |
| Smoking | 625 (7.66%) | 626 (7.66%) | 0.998 |
| Drinking status |  |  | 0.213 |
| ever | 52 (2.21%) | 235 (2.88%) |  |
| current | 343 (14.58%) | 1171 (14.34%) |  |
| Never | 1958 (83.21%) | 6762 (82.79%) |  |

Table S4. Stratified associations between GHR and the progression from normoglycemia to pre-DM by age, sex, SBP, physical activity, smoking, and drinking.

| Characteristic | No of participants | HR (95%CI) P value P for interaction |
| --- | --- | --- |
| Age(years) |  | 0.6887 |
| <30 | 816 | 0.988 (0.687, 1.421) 0.9472 |
| 30-40 | 4152 | 1.094 (1.041, 1.150) 0.0004 |
| 40-50 | 2346 | 1.054 (1.007, 1.104) 0.0237 |
| ≥50 | 854 | 1.064 (1.004, 1.129) 0.0376 |
| Sex |  | 0.3707 |
| Male | 4988 | 1.104 (0.989, 1.233) 0.0770 |
| Female | 3170 | 1.046 (1.012, 1.082) 0.0081 |
| SBP (mmHg) |  | 0.8198 |
| <140 | 7653 | 1.054 (1.019, 1.091) 0.0024 |
| ≥140 | 515 | 1.045 (0.968, 1.127) 0.2581 |
| DBP (mmHg) |  | 0.5522 |
| <90 | 7554 | 1.057 (1.022, 1.093) 0.0014 |
| ≥90 | 614 | 1.030 (0.950, 1.117) 0.4753 |
| Physical Activity |  | 0.8824 |
| **Sedentary** | 1715 | 1.060 (1.001, 1.122) 0.0472 |
| **Light activity** | 3113 | 1.042 (0.992, 1.094) 0.1042 |
| Moderate activity | 2622 | 1.052 (0.998, 1.109) 0.0584 |
| Vigorous activity | 718 | 1.090 (0.980, 1.213) 0.1128 |
| Smoking |  | 0.8592 |
| No | 596 | 1.065 (1.031, 1.100) 0.0002 |
| Yes | 7533 | 1.057 (0.978, 1.143) 0.1639 |
| Drinking |  | 0.4712 |
| Never | 235 | 1.137 (0.973, 1.329) 0.1054 |
| Current | 1171 | 1.025 (0.954, 1.101) 0.5075 |
| Ever | 6762 | 1.058 (1.021, 1.095) 0.0016 |

Note 1: Above model adjusted for sex, age, drinking status, ALT, TG, FPG, physical activity,DBP, smoking status, Scr, AST, hypertension, and SBP.

Note 2: In each case, the model is not adjusted for the stratification variable.

HR, Hazard ratios; CI: confidence, Ref: reference.

Table S5 Comparison of baseline characteristics between participants with GHR <24.37 and GHR ≥24.37.

| GHR group |  | <24.37 | >=24.37 | P-value |
| --- | --- | --- | --- | --- |
| N |  | 4660 | 3508 |  |
| Age(years) |  | 40.80 ± 8.58 | 42.38 ± 8.35 | <0.001 |
| SBP (mmHg) |  | 113.41 ± 11.46 | 120.36 ± 12.19 | <0.001 |
| DBP (mmHg) |  | 73.78 ± 7.63 | 78.34 ± 7.77 | <0.001 |
| BMI (kg/m^2^) |  | 24.68 ± 3.30 | 27.45 ± 3.71 | <0.001 |
| TC (mg/dL) |  | 191.59 ± 34.63 | 203.62 ± 37.70 | <0.001 |
| LDL-c(mg/dL) |  | 116.69 ± 32.00 | 130.90 ± 33.95 | <0.001 |
| HDL-c(mg/dL) |  | 55.08 ± 13.11 | 42.00 ± 9.35 | <0.001 |
| TG (mg/dL) |  | 99.24 ± 45.90 | 155.87 ± 96.60 | <0.001 |
| AST(u/L) |  | 26.43 ± 11.04 | 32.07 ± 10.73 | <0.001 |
| ALT(u/L) |  | 31.45 ± 12.11 | 47.85 ± 20.91 | <0.001 |
| CRP (mg/dL) |  | 2.10 ± 5.27 | 2.52 ± 4.04 | <0.001 |
| GGT |  | 20.44 ± 6.19 | 44.33 ± 19.16 | <0.001 |
| Scr(mmol/L) |  | 69.71 ± 15.34 | 70.17 ± 15.65 | 0.185 |
| Sex |  |  |  | <0.001 |
| Female |  | 1955 (41.95%) | 215 (6.13%) |  |
| Male |  | 2705 (58.05%) | 3293 (93.87%) |  |
| Hypertension |  |  |  | <0.001 |
| No |  | 4379 (93.97%) | 3035 (86.52%) |  |
| Yes |  | 281 (6.03%) | 473 (13.48%) |  |
| smoking |  |  |  | <0.001 |
| No |  | 4344 (93.22%) | 3198 (91.16%) |  |
| Yes |  | 316 (6.78%) | 310 (8.84%) |  |
| Physical Activity |  |  |  | <0.001 |
| **Sedentary** |  | 844 (18.11%) | 871 (24.83%) |  |
| **Light activity** |  | 1748 (37.51%) | 1365 (38.91%) |  |
| Moderate activity |  | 1597 (34.27%) | 1025 (29.22%) |  |
| Vigorous activity |  | 471 (10.11%) | 247 (7.04%) |  |
| Drinking status |  |  |  | 0.161 |
| Never |  | 140 (3.00%) | 95 (2.71%) |  |
| current |  | 640 (13.73%) | 531 (15.14%) |  |
| ever |  | 3880 (83.26%) | 2882 (82.16%) |  |

Abbreviations: GGT, γ-glutamyl transferase; GHR, the ratio of γ-glutamyl transferase to high-density lipoprotein cholesterol; HbA1c,**Hemoglobin A1c;** LDL-c, low-density lipid cholesterol; FPG, fasting plasma glucose; SBP, systolic blood pressure; TG triglyceride; BMI, body mass index; TC, total cholesterol, ALT, alanine aminotransferase; DBP, diastolic blood pressure; HDL-c, high-density lipoprotein cholesterol; AST aspartate aminotransferase; Scr, serum creatinine.
